# Monetary Incentives Increase Metacognitive Confidence in Source Memory Performance in Patients With Schizophrenia

**DOI:** 10.3389/fpsyt.2020.00725

**Published:** 2020-07-29

**Authors:** Remigiusz Szczepanowski, Ewelina Cichoń, Aleksandra Helena Pasieczna, Łukasz Gawęda, Joanna Rosińczuk

**Affiliations:** ^1^ Department of Public Health, Faculty of Health Sciences, Wroclaw Medical University, Wrocław, Poland; ^2^ Department of Psychology, WSB University in Torun, Torun, Poland; ^3^ Department of Psychology, Faculty of Education, University of Lower Silesia, Wrocław, Poland; ^4^ Department of Finance and Accounting, Kozminski University, Wrocław, Poland; ^5^ Institute of Psychology, Polish Academy of Sciences, Warsaw, Poland; ^6^ Department of Clinical Nursing, Faculty of Health Sciences, Wroclaw Medical University, Wrocław, Poland

**Keywords:** metacognition, memory, schizophrenia, delusions, source memory task

## Abstract

**Background:**

Contemporary psychiatric research focuses its attention on the patient’s dysfunction of metacognition in relation to the basic cognitive processes of mental activity. The current study investigated dysfunctional metacognition in relation to self-monitoring of memory in patients diagnosed with schizophrenia. Dysfunctions in metacognition were examined by focusing on two types of metacognitive measures: post-decision wagering (PDW) scale and confidence ratings (CR) scale (CR).

**Objectives:**

The research employed an action-memory task that required patients with schizophrenia (N = 39) and healthy controls (N = 50) to evaluate their metacognition by categorizing self-monitoring actions (imagined vs. performed actions) either with PDW or CR. It was hypothesized that metacognition in self-monitoring activity in patients diagnosed with schizophrenia is improved by imaginary monetary incentives.

**Material and Methods:**

To test this hypothesis, participants were asked to memorize actions either performed or imagined during the first phase of the experiment. The second phase was to identify previous actions as performed, imagined or new, and then to express confidence using two measures of metacognition (CR or PDW scales) that were randomly allocated to participants.

**Results:**

Our study showed reduced performance in the action memory task for patients with schizophrenia, although there were no group differences in confidence measures when assessing self-monitoring actions. In particular, irrespective of the diagnosis, no differences in confidence measures for correct responses were found in the case of the PDW and CR scales. We also observed that metacognitive judgements were more accurate for incorrect responses when both groups used monetary incentives to reveal their metacognition.

**Conclusions:**

Our findings suggest that monetary incentives improve accuracy of metacognition among both patients and healthy controls. This accuracy-enhancing effect of monetary incentives on metacognition was possibly a result of motivational processes, including aversion to loss. The paper discusses the potential application of PDW in therapeutic metacognitive training for patients with schizophrenia.

## Introduction

Recent clinical research has adopted a metacognitive approach to treating schizophrenia spectrum disorders (1, 2). The notion of “metacognition” *per se* describes cognitive processes that are linked with activation of “thinking about one’s own thinking”, by which individuals can reflect upon (monitor) their own internal mental states and apply their knowledge to evaluate and regulate (control) their own mental states (3). This theoretical approach claims that abnormality of higher-order processes and knowledge is responsible for dysfunctional regulation of the primary cognitive processes (i.e., memory and other cognitive functions) (3) and may lead to severe mental disorders. For the sake of brevity, abnormality of metacognition relates to impairments in control and monitoring and higher-order knowledge structures (e.g., beliefs) that together regulate storage and acquisition of information from different modalities. Given this theoretical view, clinicians may identify cognitive causes that lead to the formation and persistence of psychopathological symptoms in a variety of mental disorders, including psychotic disorders, schizophrenia, or anxiety disorders (1–6).

In fact, the dysfunctional operations that underlie metacognition are cognitive biases and include disturbances in accuracy of metacognition, impaired sense of confidence in responses, or dysfunctional cognitive strategies in regulating one’s own behavior (4). A thorough description of abnormal metacognitive processes and knowledge structures is presented by Moritz and Woodward (7) [see also (4, 8)]. For example, in the case of the schizophrenia population, cognitive biases such as overconfidence are considered the basic driving mechanisms that perpetuate false judgments (2). In addition, several studies have also shown that inadequate confidence related to one’s own experiences may preclude regulative mistrust in faulty decisions. In turn, this may lead to extreme beliefs and distortions in perceiving reality (e.g., in psychosis) (9) and prevent alternative explanations due to the holding of false information with strong conviction (knowledge corruption) (10). Several studies have shown that patients with schizophrenia are not only overconfident in false memories, but they are also underconfident in correct responses (10–13).

It is worth mentioning that the available theoretical concepts have advanced and refined the view on the abnormality of metacognition and the basic cognitive processes in schizophrenia. For instance, jumping to conclusions bias (JTC) or liberal acceptance bias may be useful in explaining this response pattern. Moritz and Woodward (14) suggested that patients with schizophrenia make memory decisions confidently and rapidly by relying more on the mere familiarity of information. This tendency promotes highly confident errors in patients and also, to some extent, less confidence in correct responses. On the other hand, healthy individuals apply more rigorous strategies based on the reluctance to fully accept answers based on partial evidence (7). Therefore, healthy control participants require rich cue information to ensure high confidence in their responses. This account suggests that if patients with schizophrenia adopted more vigilant strategies based on the detection of more cues (7), their confidence in errors might be reduced, and their confidence in correct responses might be enhanced.

From the experimental perspective, there are several measures of metacognition in which participants assess the accuracy of their first-order discriminations. Typically, confidence (CR) represents metacognitive judgments that describe a participant’s confidence in how certain they are about processing a given stimulus or how accurate their own responses were in a given task (15). On the other hand, in a post-decision wagering (PDW) task, participants use economic categorization to reveal their metaknowledge about first-order cognitions (16). In particular, they are asked to discriminate an item (e.g., a memorized or seen object) and then wager first-order discrimination decisions. Importantly, participants are told that correct wagers are rewarded by imaginary or real earnings, while incorrect wagers are deducted from earnings. In this fashion, they start to believe that they are playing a sort of gambling game that can lead to losses (17). For instance, Clifford, Arabzadeh, and Harris (18, 19) demonstrated that the pay-off matrix of PDW can favor a particular gambling strategy, e.g., participants always tend to bet high. The response criterion of wagering can also be biased strongly by the loss aversion that is induced by monetary incentives (20). In fact, loss aversion may result in a precautionary strategy that often is based on low wager ratings even when participants are aware of the stimuli but are not fully confident in their first-order discriminations (21). Thus, the effect of loss aversion induced by monetary incentives seems to urge caution in the subjective assessment of patients’ erroneous memories.

Indeed, our previous empirical study on a healthy population indicated that metacognitive judgments were more accurate when participants used the PDW scale rather than the confidence ratings (CR) scale in an action memory task (22). In this study, we required participants to perform or imagine physical actions and then distinguish performed and imagined memories. Finally, to reveal metacognitive judgments, participants rated their confidence in responses with CR (the numerical scale) or with imaginary monetary wagers (PDW) that engaged a more vigilant strategy based on loss aversion. Our previous research demonstrated that imaginary money resulted in more accurate metacognition because healthy participants who categorized their certainty with imaginary monetary wagers expressed lower confidence in self-monitoring errors (i.e., “misattributed imagined-as-performed” responses) than in responses expressed with the CR scale (22). Thus, monetary incentives contributed to better metacognition accuracy in the case of misattribution errors (i.e., misattributed imagined-as-performed actions; misattributed performed-as-imagined actions). These findings, in turn, may suggest that metacognition driven by economical categorization may reduce overconfidence in faulty decisions and beliefs in patients with schizophrenia.

It is important to mention that basic cognitive activity such as source-monitoring (e.g., the ability to distinguish the origins of information, e.g., perceived and imagined memories presented in an action memory task) may be fundamental in establishing adequate cognitive functioning in the real world that leads to adaptive behavior and effective decision-making (23). In fact, an individual’s capacity to discriminate the sources of mental experience serves as a theoretical background to explain reality distortions in a variety of mental disorders, such as hallucinations (9, 24, 25) or self-disturbances (24, 25). For instance, Gawęda and colleagues (9, 26) employed an analogical action memory paradigm to investigate self-monitoring processes in patients with schizophrenia. It was shown that patients diagnosed with schizophrenia committed more errors in the action memory task than healthy participants. Moreover, since the experimenters measured patients’ metacognition with the CR scale, they observed that patients expressed overconfidence when they committed self-monitoring errors (9). Thus, the overconfidence related to self-monitoring errors observed in this study suggests that abnormality of metacognition may be involved in impairments in cognitive functioning in schizophrenia (9–14, 27).

Taken together, the above outcomes and premises raise an important question for psychiatry research as to whether metacognition based on monetary incentives (i.e., wagering with imaginary money) improves the accuracy of metacognition in individuals diagnosed with schizophrenia who perform self-monitoring of their own memory. To examine this claim, patients and healthy controls undertook rating tasks with two metacognition assessments (CRs vs. PDW) to evaluate performance in self-monitoring actions induced by an action memory paradigm. This experimental condition represents a typical metamemory paradigm that is intended to investigate subjective memory functioning and confidence (10, 26, 28). In fact, as opposed to healthy controls and non-schizophrenia psychiatric controls, several metamemory studies provide substantial evidence for the “*overconfidence effect*” because patients suffering from schizophrenia display robust overconfidence in memory errors and moderate effects of underconfidence in correct responses (26, 29, 30). Moreover, in order to investigate the relationships between confidence and psychosis in clinical populations, most of these memory studies use a conventional confidence rating scale that asks patients to reveal their confidence in their metamemory on this scale (9, 29, 31, 32). Yet, to the best of our knowledge no empirical study has used the PDW scale to investigate accuracy of metacognition in patients with schizophrenia. Determining whether patients with schizophrenia are accurately aware of their memory performance when stimulated with monetary incentives might be a real implication for psychological interventions. In the non-clinical population, judgments based on PDW when evaluating source monitoring performance were found to be more accurate than those based on the CR scale (22). In the present study, it was therefore expected that patients with schizophrenia would evaluate their subjective memory functioning with PDW more objectively, thus diminishing the presence of the “overconfidence effect”.

## Material and Methods

### Participants

Eighty-nine individuals (50 healthy and 39 diagnosed as patients with schizophrenia; 10 inpatients, 29 outpatients) participated in this study. Patients fulfilled ICD-10 criteria for schizophrenia, as determined by an experienced psychiatrist at “Zielone Wzgórza” Social Welfare Home in Rościszów, Maria Med Center for Psychiatry and Psychology in Lubin, “Sudeckie Centrum Zdrowia” Non-public Healthcare Centre in Pieszyce, and Lower Silesian Mental Health Centre in Wrocław. Additionally, before testing, the psychiatrist’s clinical diagnoses were confirmed by The Mini-International Neuropsychiatric Interview (33), which follows DSM-IV criteria. Exclusion criteria were alcohol and/or drug abuse or any form of documented or suspected neurological diseases. All patients were receiving atypical neuroleptic medication and were stable at the time of testing. **Table 1** presents the equivalents of mean daily antipsychotics doses to chlorpromazine (CPZ) that were calculated for each group of patients based on formulas relevant to defined daily doses (DDDs) presented by the World Health Organization’s Collaborative Center for Drug Statistics Methodology (34). At the time of their participation, all of the schizophrenia participants were in treatment. Fifty healthy participants with a declared lack of life-time prevalence of any mental disorders served as controls. Healthy controls were recruited from students of Psychology at SWPS University of Social Sciences and Humanities in Wroclaw. The students responded to the announcement about the study *via* the university’s internal messaging system. The computer experiment was carried out in the laboratory room. Every participant took part in the study after informed consent was obtained. The study received the approval of the local ethical committees. The experiment was repeated three times. Two experiments were conducted on non-clinical populations (22, 35), and the third study on a clinical population of patients with schizophrenia was a part of an undergraduate research project in the Department of Psychology at the University of Lower Silesia (36).

**Table 1:** Demographic characteristics of the group of patients with schizophrenia and healthy control.

Demographics		Healthy N = 50		Patients N = 39		Statistics
		CR	PDW	CR	PDW	
Gender	Males/females	8/17	5/20	10/9	15/5	χ2(3) = 15.729, p = 0.001
		17	20	9	5	**Statistics**
		χ2(1) = 0.936, p = 0.333		χ2(1) = 2.12, p = 0.146		
Age		31.2 (7.94)	26.3 (7.73)	39.0 (16.49)	37.8 (11.40)	F = 16.767, p < 0.001
Years of sickness		–	–	14.0 (11.61)	17.1 (8.97)	*F* = 0.739, *p* > 0.3
In/outpatients^a^		–	–	48.28	50.0	χ2 = 0.01, *p* > 0.9
CPZ equivalent dosage^b^ (mg/day)		–	–	846.26 (1478.14)	807.29 (790.27)	*F* = 0.01, *p* > 0.9

^a^percent of outpatients within sub-group; ^b^dosage of the medication in chlorpromazine equivalent (34).

We split the populations’ samples according to the response mode (half of the participants were randomly assigned to the PDW scale and half to the CR scale) and expected group differences in that higher metacognition accuracy would be present for patients using economic-based categorization (PDW scale) as opposed to categorization based only on beliefs about one’s own cognitions (CR scale).

### Psychopathology Assessment of Schizophrenia

Psychopathology in patients was assessed with a semi-structured interview: Scale for the Assessment of Positive Symptoms (SAPS) (37) and Scale for the Assessment of Negative Symptoms (SANS) (38).

SANS includes 25 items related to negative symptoms. Raters assess each item using a 6-point Likert scale (from 0 to 5). Higher scores indicate higher symptom severity. The scale evaluates five domains: affective blunting, alogia, avolition/apathy, anhedonia/asociality, and attention (38–41). We assessed negative symptoms with the SANS scale by looking at these five subscales (39). The SAPS measures positive symptoms and allowed us to identify hallucinations, delusions, positive formal thought disorders and bizarre behavior (37). This scale consists of 34 items assessed on a 6-point scale (from 0 to 5). Items are categorized into four domains: hallucinations, delusions, bizarre behavior, and positive formal thought disorders (37, 41).

Thus, these two scales provide comprehensive measures of the symptoms of schizophrenia. It is important to note that SAPS and SANS scores were available for only 30 patients because nine patients took part in the action memory task but were then unavailable due to unpredicted discharge from the ward (see **Table 2**). There were no differences in SAPS, *F*(1,28) = 0.36; *p* = .851 and SANS scores *F*(1,28) = 2.73; *p* = .109 between patients with schizophrenia that were randomly assigned to PDW and CR conditions.

**Table 2:** Psychopathological characteristics of the CR and PDW groups and group differences in SAPS and SANS scores among patients with schizophrenia.

		Schizophrenia (N = 30)*				
		CR (n = 14)		PDW (n = 16)		
SANS		Mean	SD	Mean	SD	
	Affective blunting	21.21	9.98	19.37	8.82	
	Alogia	11.50	6.31	12.50	5.97	
	Avolition-Apathy	8.00	5.22	10.31	5.86	
	Anhedonia-Asociality	12.79	7.81	12.56	4.52	
	Attentional Impairment	7.00	4.40	7.50	4.10	
	**SANS total score**	60.43	28.82	62.25	23.81	F(1,28) = 0.36, p = .851
**SAPS**						
	Hallucinations	9.29	9.19	13.94	11.94	
	Delusions	17.86	16.21	24.94	23.91	
	Bizarre behavior	6.36	4.89	9.81	6.18	
	Positive formal thought disorder	8.00	11.52	20.19	13.54	
	**SAPS total score**	41.50	38.82	68.87	50.11	F(1,28) = 2.73, p = .109

*SAPS and SANS scores were available for only 30 patients because nine patients took part in the action memory task but were then unavailable due to unpredicted discharge from the ward.

### Action Memory Task

Participants undertook an action memory task that is commonly employed in studies of self-monitoring deficits in schizophrenia (9) and obsessive-compulsive disorder (42) populations. The procedure of this study consisted of two phases. In the learning phase, participants were asked to imagine or perform presented actions in accordance with verbal instructions displayed on a computer screen. Instructions set in a green frame had to be performed by the participants, whereas action instructions set in a red frame had to be imagined but not performed. Before the experiment, all participants were instructed that they would have to recall the presented actions and distinguish whether they had been imagined or performed by them. The participants performed a short practice trial to become familiar with the task requirements. In the main learning phase, we used 19 items for the participants to perform and 19 items to imagine. Each instruction with information about the action was displayed on the computer screen once for 10 s. In the second phase of this study, 38 verbal instructions of the learning-phase items were presented along with 20 new action instructions. The number of correct responses given by participants ranged from 0 to 58. The maximum number of possible false alarms was 20 (20 new items recognized as performed or imagined actions); the maximum number of possible forgotten actions was 38 (presented actions: imagined or performed recognized as new); the number of monitoring errors ranged from 0 to 38 because this was the sum of “imagined actions recognized as performed” (maximum of 19) and “performed actions remembered as imagined” (maximum of 19). Moreover, in order to prevent physical matching, the items were presented in different fonts and placed in different locations on the screen than those used for the earlier items. At the end of the experiment, participants were asked to respond to whether a given instruction was new or had been presented in the learning phase as a performed or imagined action; they were then required to express their metacognition in a self-monitoring activity based on CR or PDW, each of which was randomly assigned to the selected group.

### Subjective Measures in Self-Monitoring

**Figure 1:**
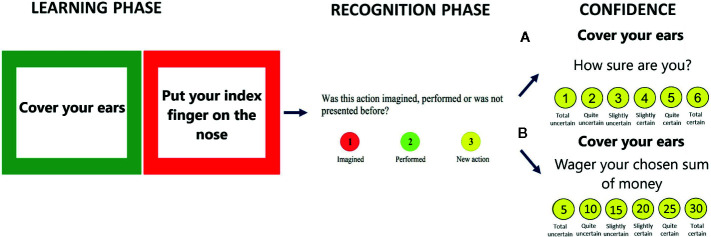
The action memory task with confidence assessment: Demographic characteristics of the group of patients with schizophrenia and healthy control **(A)** CR scale or **(B)** PDW scale.

This experiment used two measures of metacognition that were randomized across participants. The participants were asked to rate their certainty in recognizing responses from the action memory task by choosing the numerical keys on the keyboard (from 1 to 6). The first scale was a CR scale with six levels expressed as such 1—Totally uncertain, 2—Quite uncertain, 3—Slightly uncertain, 4—Slightly certain, 5—Quite certain, and 6—Totally certain. The second scale was adapted from the PDW (15, 43, 44) task and consisted of asking participants to express their confidence as wagers in imagined money. No reward for the performance of the task was in fact awarded. Hence, wagers of 5, 10, 15, 20, 25, and 30 PLN (1 PLN is around 0.24 EUR) were used in this experiment (see **Figure 1**). Participants were informed that the study was a sort of gambling game with imaginary earnings. They were asked to wager an imaginary amount of money that they won when they made correct discriminations. When an answer was wrong, the participants were told that the wagered amount would be lost.

### Statistical Analyses

The observed performance parameters were old/new recognition (imagined as imagined, performed as performed, new as new), false alarms (new as performed or imagined), forgotten (performed or imagined as new), and self-monitoring (imagined as performed, performed as imagined). Similarly, we calculated the metacognition accuracy on the basis of the confidence levels.

The participants’ responses were collected, and in the next step we examined the influence of the diagnosis (healthy vs. unhealthy) and the type of metacognitive scale (CR vs. PDW) on performance in the action memory task. To do so, correct and incorrect item recognitions (old/new items) and correct and incorrect source-monitoring recognitions were calculated. The effects of these variables on metacognition accuracy were investigated by two-way analysis of covariance (two-way ANCOVA). The main effects of the diagnosis and metacognitive scale and the interaction effects on old/new item recognition and source-monitoring responses were then examined. Age was taken as a covariate in the ANCOVA analysis. All calculations were performed with SPSS software and a significance level of 0.05 was established. Our sample included 89 participants. For the purpose of this study, we calculated the required sample size using G*Power software (v3.1) beforehand. We simulated F tests for ANCOVA, including fixed effects, main effects, and interactions with the following assumptions: small effect size, α err prob = 0.05, power (1-β err prob) = 0.80, df = 1, Number of groups = 4, Number of covariates = 0. For these parameters, the simulation revealed that a total sample size of 73 participants was needed.

## Results

### Sociodemographic Characteristics

Demographic and psychopathological characteristics are presented in **Table 1**. There were differences in age between the schizophrenia patients (*M* = 38.41, *SD* = 13.94) and the healthy controls (*M* = 28.74, *SD* = 8.14), *F*(1, 87) = 16.767, *p* < 0.001, partial η^2^ = 0.162. The age variable was controlled for in the analysis. The group of patients included 25 males and 14 females, although the gender difference was not significant, χ2(1) = 3.10, *p* = 0.078, as opposed to the healthy controls (13 males/37 females), χ2(1) = 11.52, *p* < 0.001. In the group of patients, 10 males used the CR scale (9 females) and 15 males used the PDW scale (5 females). The gender association with the scale was not significant in the group of patients, χ2(1) = 2.12, *p* = 0.146. In the control group, 8 males used the CR scale (17 females) and 5 males used the PDW scale (20 females). No significant gender association with type of scale was found in the group of healthy controls, χ2(1) = 0.936, *p* = .333. The participants were assigned to four categories: 1) healthy controls using the CR scale; 2) healthy controls using the PDW scale; 3) patients using the CR scale; 4) patients using the PDW scale. There was a significant association between gender and participants’ category, χ2(3) = 15.729, *p* = 0.001.

### Performance: The Effect of Diagnosis (Patients With Schizophrenia vs. Healthy Controls) on Old/New Recognition and Self-Monitoring Errors

The results indicated that the main effect of diagnosis on performance was significant. In the case of old/new recognition, healthy participants demonstrated better performance than patients with schizophrenia, *F*(1, 84) = 9.37, *p* = .003; partial η^2^ = 0.10. For the false alarms parameter (new items recognized as old), there was no difference between the healthy controls and the patient group, *F*(1, 84) = 2.52, *p* = 0.116; partial η2 = 0.03. In the case of forgetting (old items recognized as new), there was no significant effect of diagnosis on performance, *F*(1, 84) = 2.23, *p* = 0.139; partial η2 = 0.03.

For the self-monitoring (imagined actions recognized as performed) responses, we also observed that healthy participants (M = 2.36, SD = 1.88) recognized the information source better than the patients did (M = 4.28, SD = 3.68), *F* (1, 85) = 10.08, *p* = .019, and partial η^2^ = 0.064. In addition, there was no group difference with regards to performed actions recognized as imagined, *F*(1, 84) = 0.188, *p* = 0.666; partial η2 = 0.002. The results are presented in **Table 3**.

**Table 3:** The main effect of diagnosis (schizophrenia vs. healthy controls) on performance.

		Schizophrenia		Healthy participants		Statistics		
		M	SD	M	SD	*F*	p	partial η^2^
**The main effect of diagnosis**	old/new hits	34.56	9.85	42.06	6.20	9.37	.003	0.10
	false alarms	5.72	5.78	4.40	2.00	2.52	.116	0.03
	forgotten	9.28	6.39	6.00	3.82	2.23	.139	0.03
** **	***Self-monitoring errors***							
	Imagined as performed	4.28	3.68	2.36	1.88	5.71	.019	0.06
	Performed as imagined	3.18	3.28	2.86	2.16	0.28	0.60	0.00

### Performance: The Effect of Type of Scale (CR vs. PDW) on Old/New Recognition and Self-Monitoring Errors

In the next step of analysis, we examined the main effect of the type of metacognitive scale (CR or PDW) on performance. In the case of old/new recognition, we did not find any significant difference between the CR and PDW measures, *F*(1, 84) = 0.001, *p* = 0.98; partial η2 = 0.000. For the false alarm responses, there was no significant effect of metaknowledge, *F*(1, 84) = 0.43, *p* = 0.514; partial η2 = 0.005. In the case of forgetting, the difference between the CR and PDW groups was also not significant, *F*(1, 84) = 0.11, *p* = 0.739; partial η2 = 0.001. For self-monitoring, in terms of both imagined actions recognized as performed *F*(1, 84) = 0.251, *p* = 0.618; partial η2 = 0.003 and performed actions recognized as imagined, *F*(1, 84) = 0.804, *p* = 0.373; partial η2 = 0.009, the difference between the CR and PDW groups was not significant (see **Table 4**).

**Table 4:** The main effect of type of scale (CR vs. PDW) on performance.

		CR group			PDW group		Statistics		
		M		SD	M	SD	*F*	*p*	partial η^2^
The main effect of type of scale	Old/new hits	39.04		9.18	38.50	8.47	0.001	0.980	0.00
	False alarms	5.20		4.61	4.76	3.64	0.43	0.514	0.005
	Forgotten	7.50		5.29	7.38	5.43	0.11	0.739	0.001
	Self-monitoring errors							
	Imagined as performed	3.41		2.72	3.00	3.19	0.251	0.618	0.003
	Performed as imagined	2.75		2.74	3.24	2.66	0.804	0.373	0.009

### Performance: The Interaction Effect of Diagnosis (Healthy vs. Patients With Schizophrenia) and Type of Scale (CR vs. PDW) on Old/New Recognition and Self-Monitoring Errors

The interaction effects between the mental disorder and the measures of confidence are presented in **Table 4**. There was no significant interaction effect for old/new recognition, *F*(1, 84) = 0.057, *p* = 0.812; partial η2 = 0.001; false alarms, *F*(1, 84) = 0.202, *p* = 0.655; partial η2 = 0.002; forgetting, *F*(1, 84) = 0.041, *p* = 0.839; partial η2 = 0.000; or self-monitoring errors: performed recognized as imagined, *F*(1,84) = 0.159, *p* = 0.691; partial η2 = 0.002; imagined recognized as performed, *F*(1,84) = 0.017, *p* = 0.896; partial η2 = 0.000 (see **Table 5**).

**Table 5:** The interaction effect of type of scale (CR vs. PDW) and diagnosis (patients with schizophrenia vs. healthy controls) on performance.

			Schizophrenia		Healthy group		Statistics		
			M	SD	M	SD	F	*p*	partial η^2^
The interaction effect of diagnosis and scale	Old/new hits	CR	34.21	9.80	41.76	5.54	0.057	0.812	0.001
		PDW	34.90	10.13	42.36	6.89			
	False alarms	CR	6.21	6.54	4.44	2.14	0.202	0.655	0.002
		PDW	5.25	5.08	4.36	1.89			
	Forgotten	CR	9.32	6.65	6.12	3.53	0.041	0.839	0.000
		PDW	9.25	6.31	5.88	4.17			
	*Self-monitoring errors*								
	Imagined as performed	CR	4.42	3.42	2.64	1.75	0.017	0.896	0.000
		PDW	4.15	4.00	2.08	2.00			
	Performed as imagined	CR	2.79	3.61	2.72	1.90 2.42	0.159	0.691	0.002
		PDW	3.55	2.98	3.00				

### Metacognition: The Effect of Diagnosis (Patients With Schizophrenia vs. Healthy Controls) on Confidence of Old/New Recognition and Self-Monitoring Errors

The following analyses were conducted to determine whether patients with schizophrenia differ from healthy controls in terms of confidence in given responses in the action memory task.

The results showed that there was no main effect of diagnosis on old/new confidence responses, *F*(1, 84) = 0.053, *p* = 0.819; partial η2 = 0.001, and false alarms confidence, *F*(1, 81) = 2.88, *p* = 0.094; partial η2 = 0.034. For the confidence responses in forgotten items there was no difference between the healthy groups and the patient groups, *F*(1, 83) = 3.50, *p* = 0.065; partial η^2^ = 0.04. The patients with schizophrenia did not differ in CR for imagined actions recognized as performed, *F*(1, 74) = 0.007, p = 0.932; partial η2 = 0.000. There was a trend, but no statistical significance difference between groups in confidence in performed-as-imagined actions was present, *F*(1, 69) = 3.85, *p* = 0.054; partial η2 = 0.05 (see **Table 6**). Note that patients with schizophrenia expressed higher confidence in misattributed performed-as-imagined actions (*M* = 4.69, *SD* = 1.34) than the healthy controls *(M* = 4.20, *SD* = 1.23).

**Table 6:** The main effect of diagnosis (patients’ group vs. healthy controls) on confidence ratings.

		Schizophrenia		Healthy controls			Statistics				
	Confidence	M	SD	M	SD		F		p		partial η2
The main effect of diagnosis	Old/new hits	5.18	0.103	5.14	0.58		0.053		0.819		0.001
	False alarms	4.48	1.49	4.06	0.88		2.88		0.094		0.034
	Forgotten	4.85	1.32	4.21	1.21		3.50		0.065		0.04
	Self-monitoring errors										
	Imagined as performed	4.41	1.76	4.44	1.27		0.007		0.932		0.000	
	Performed as imagined	4.69	1.34	4.20	1.23		3.85		0.054		0.05	

### Metacognition: The Effect of the Type of Scale (CR vs. PDW) on Confidence in Old/New Recognition and Self-Monitoring Errors

The results from the main effect of the usage of different metacognitive measures on confidence responses are presented in **Table 7**. There was no main effect of the metacognitive scale on confidence in old/new recognitions, *F*(1, 84) = 2.91, *p* = 0.092; partial η2 = 0.034. In the case of confidence produced for false alarms, there was a significant difference between participants who used the CR and PDW scales, *F*(1, 81) = 9.146, *p* = 0.003; partial η^2^ = 0.101. The results showed that lower confidence was used to assess false alarms when participants employed PDW (*M* = 3.88, *SD* = 1.27) than when they used the CR scale (*M* = 4.60, *SD* = 0.98). For confidence in forgotten items, there was also a significant difference between the CR and PDW groups, *F*(1, 83) = 5.68, *p* = 0.019; partial η^2^ = 0.064. For forgotten actions, participants also assessed their confidence as lower when they categorized metacognition with PDW (*M* = 4.17, *SD* = 1.35) as opposed to CR (*M* = 4.82, *SD* = 1.15). It turned out that the main effect of metacognition measures was also significant for confidence in terms of self-monitoring errors. In particular, for performed actions that were recognized as imagined, participants produced more confident misrecognitions using CR scale (M = 4.84, SD = 1.06) as compared to the PDW scale (M = 3.98, SD = 1.36), F(1, 69) = 9.168, *p* = 0.003; partial η^2^ = 0.117. In the case of imagined actions that were recognized as performed, participants also committed more confident misrecognitions using the CR scale (*M* = 4.89, *SD* = 1.16) as compared to the PDW scale (*M* = 3.95, *SD* = 1.67), *F*(1, 74) = 7.762, *p* = 0.007; partial η^2^ = 0.095.

**Table 7:** The main effect of type of scale (CR vs. PDW) on confidence ratings.

		CR group		PDW group		Statistics		
	Confidence	M	SD	M	SD	F	*p*	partial η^2^
The main effect of metacognition measure	Old/new hits	5.31	0.80	5.01	0.79	2.91	0.092	0.034
	False alarms	4.60	0.98	3.88	0.27	9.146	0.003	0.101
	Forgotten	4.82	1.15	4.17	1.35	5.68	0.019	0.064
	Self-monitoring errors							
	Imagined as performed	4.89	1.16	3.95	1.67	7.762	0.007	0.095
	Performed as imagined	4.84	1.06	3.98	1.36	9.168	0.003	0.117

### Metacognition: The Interaction Effect of Diagnosis (Schizophrenia vs. Healthy Controls) and Type of Scale (CR vs. PDW) on Confidence in Old/New Recognition and Self-Monitoring Errors

In the final analysis, we investigated how both the diagnosis and the type of metacognitive scale affected confidence (see **Table 8**). Between both factors there were no significant interaction effects on confidence (see **Table 8**).

**Table 8:** The interaction effect of type of scale (CR vs. PDW) and diagnosis (Schizophrenia vs. Healthy controls) on confidence ratings.

		Schizophrenia			Healthy controls		Statistics		
			M	SD	M	SD	*F*	*p*	partial η^2^
**The interaction effect of diagnosis and scale**	Old/new hits	CR	5.29	1.10	5.33	0.49	0.227	0.635	0.003
		PDW	5.07	0.98	4.95	0.62			
	False alarms	CR	4.95	1.20	4.37	0.73	0.309	0.580	0.004
		PDW	4.06	1.63	3.75	0.93			
	Forgotten	CR	5.19	1.19	4.54	1.05	0.004	0.953	0.000
		PDW	4.53	1.39	3.86	1.28			
	*Self-monitoring errors*								
	Imagined as performed	CR	4.78	1.42	4.97	0.97	0.415	0.521	0.006
		PDW	4.07	2.00	3.83	1.32			
	Performed as imagined	CR	5.13	1.27	4.68	0.91	0.066	0.798	0.001
		PDW	4.35	1.33	3.68	1.34			

## Discussion

The present study investigated dysfunctional metacognition in self-monitoring of memory in patients diagnosed with schizophrenia. Dysfunctions in metacognition were assessed with either monetary incentives (PDW scale) and the conventional measure of confidence based on the CR scale. Our study demonstrated for the first time that accuracy of metacognition about correct responses and memory errors in source-monitoring performance in patients with schizophrenia and healthy controls improved due to imaginary wagering (PDW scale). For all groups of participants, more accurate confidence responses were revealed with monetary categorizations. This effect is of particular importance for clinical practice because the accuracy of metacognition in patients with schizophrenia improved when using the PDW as opposed to the CR scale. Our research therefore suggests that mistaken trust in self-monitoring actions may be reduced by engaging the wagering strategy that induced motivational processes and aversion to loss in patients with schizophrenia and healthy participants. The findings are in the line with previous research investigating metacognitive processes of control and monitoring in a general knowledge task with monetary incentive cues to encourage participants’ accurate answers (45). Although schizophrenia patients had defective metacognition in this study, i.e., their subjective accuracy of correctness was impaired, their modulation of control (response criteria adjustments) was intact when a gambling strategy was involved that required them to bet a small amount of money on each response (45). Thus, this study suggests that patients with schizophrenia could improve their performance when encouraged to use monetary incentives (45). Similarly, our study shows that patients with schizophrenia can improve their metacognitive accuracy in self-monitoring tasks by engaging a gambling strategy to assess their confidence subjectively.

In addition, our research shows that patients with schizophrenia displayed lower performance than healthy subjects with respect to old/new recognition as well as self-monitoring responses. The patients with schizophrenia misremembered imagined actions as having been performed more often than healthy controls, but not *vice versa.* In fact, these results confirm observations from previous self-monitoring studies (9, 46) that indicated a specific cognitive bias pattern regarding self-monitoring errors in patients with schizophrenia who presented more misremembered imagined actions than were really performed, but not the reverse. This outcome may be explained by the hypothesis concerning *over-perceptualization* (47) that was proposed within the neuroanatomical model by Allen and colleagues [(47), see also (27)], which describes a network of brain areas and their respective contributions to the hallucinatory experience. The core of this model is hyperactivation of the secondary sensory cortex among patients while they are experiencing hallucinations. This account assumes that bottom-up dysfunctions through over-activation in the secondary and primary sensory cortices may lead to the experience of vivid perceptions in the absence of sensory stimuli (27, 47). The specific pattern of performance in patients with schizophrenia who more often misremembered imagined actions as having been performed may be explained to some extent by the account that demonstrates more vivid imagery in patients with schizophrenia (48).

Moreover, our study demonstrated that both the PDW and CR measures did not affect performance in the action memory task. The non-significant interaction effects from the ANCOVAs indicted that patients with schizophrenia achieved lower performance in self-monitoring regardless of the measure of metacognition. We also found that patients with schizophrenia showed a tendency (*p* = 0.054) to express over-confidence in self-monitoring errors (performed as imagined) for both metacognition-measurement scales. In fact, the overconfidence phenomenon for action self-monitoring errors has been found in schizophrenia in several previous studies (11, 28, 32). Contrary to what was hypothesized, our results suggested a lack of impairment in metacognition among patients with schizophrenia. Thus, by using the metacognitive judgments of PDW or CR, patients with schizophrenia were found to evaluate source memory performance accurately, which suggests a dissociation between preserved metacognition and altered source-monitoring abilities. Interestingly, similar findings indicating preserved metacognitive functions which do not correspond to impaired memory performance were observed in patients with Alzheimer’s disease (49), people with symptoms of amnesia (50), and in other metamemory studies in schizophrenia patients (51, 52). For example, Souchay et al. (51) examined metamemory and memory performance in patients with schizophrenia and control subjects by using a Feeling of Knowing (FOK)^1^ task on episodic memory information. The results of Souchay et al. (44) clearly suggested a dissociation between impaired memory and preserved metacognitive ability to predict recognition performance accurately in patients with schizophrenia. Another study by Bacon at al. (52) investigated knowledge about one’s own memory capability in patients with schizophrenia. Again, there was a clear dissociation between impaired semantic memory and accurate metacognition expressed with CR with regard to recollection processes in patients. However, another measure of metaknowledge based on FOK judgments that was dependent on information accessibility was significantly impaired in patients with schizophrenia. Our results confirm these findings, since preserved metacognitive ability in schizophrenia in relation to source-monitoring performance was about correctness of information from source-monitoring in the present study. This, in turn, might suggest that patients with schizophrenia (like healthy controls) are aware of source-monitoring performance when information processing is induced by a scale (PDW vs. CR).

Irrespective of the diagnosis categorization, our analysis indicated that accuracy of metacognition was better for PDW measure engaging the gambling strategy as compared to the conventional CR measure. In the case of using metacognitive judgments evoked by PDW scale, for both populations, we observed lower confidence expressed in all types of erroneous responses, such as false alarms, forgotten actions, and self-monitoring errors. Thus, patients presented enhanced metacognitive accuracy that was activated with the PDW scale. The demonstration of preserved metacognitive functions may have implications for the treatment of delusional convictions in patients with schizophrenia. Delusions are commonly defined as false beliefs that are maintained by patients with strong convictions (56). Since overconfidence is usually associated with delusional symptoms in schizophrenia (57), the reduction of such a bias may influence their treatment outcomes. Indeed, recent research on metacognitive intervention has indicated that the prognosis is good for the treatment of patients with schizophrenia who are aware of their memory function deficits (2, 58–60). Our study strongly suggests that monetary incentives may enhance the accuracy of metamemory in patients with schizophrenia and, therefore, may to some extent weaken their strong convictions in inaccurate inferences. Thus, taken together, the results of this study and our previous findings (22) indicate that encouraging patients’ own assessments of performance with monetary incentives may be applicable to the treatment of metacognition in terms of reducing the severity of delusions in schizophrenia.

Several studies on metamemory in people with schizophrenia suggest that inaccurate metacognition arises from the fact that patients (similarly to healthy controls) are not aware of their deficits in some domains of everyday functioning or are not aware of their own skills and abilities (57, 61). It is well documented in experimental studies on motivation in the general population that monetary incentives are substantial ingredients of motivation that activate an individual’s internalized drive to take action (62) and may increase metacognitive awareness and/or first-order discrimination (15). In the same vein, monetary incentives may act as stimuli for better first-order performance in people with schizophrenia by concurrently enhancing second-order discriminations, i.e., the accuracy of metacognition. On the other hand, from the cognitive perspective, post-wagering may be subjected to a variety of confounding factors (including loss aversion) (16) that result in a conservative wagering strategy that involves systematically using smaller wagers, even though participants are aware of the stimuli (17). Regardless of these conceptual issues, taking into account models of metacognitive regulation (63, 64) and phenomenology of delusional beliefs (57), the accuracy of preserved metamemory that is dissociated from source memory performance may still be increased and may have therapeutic effects in terms of weakening delusional inferences in schizophrenia.

Interestingly, from a cognitive perspective our results suggest that bottom-up processes involving primary structures of the brain related to basic performance (e.g., distinguishing the memory sources) are resistant to meta-knowledge manipulation. On the other hand, according to the neuroanatomical model, the psychotic experience is augmented by a weakening of top-down control driven by a variety of brain regions, e.g., the ventral anterior cingulate or the prefrontal, premotor and cerebellar cortices (45). Thus, maintenance of false experiences (e.g., hallucinations) may be caused by abnormality in control processes that do not provide effective regulation of these experiences at any given moment. According to the account presented in this study, metacognition cannot overcome errors on the primary level, although activation of metacognitive control may modify the false beliefs system when an individual uses more precise vigilant strategies to assess his/her basic performance.

Our study also has several limitations. First, whether we found the “overconfidence effect” in patients with schizophrenia may be debatable as statistical inferences relied on a *p* level of 0.054. Therefore, future replications on a larger sample are needed to increase the statistical power of our results. Second, we did not collect data relating to the educational level of patients in the demographic information. Although educational level could be a factor that may lead to poor memory functioning in schizophrenia population (64), some studies also indicate that less-educated healthy controls outperform patients with schizophrenia on memory tests (65), thus making the relations between schizophrenia and memory performance more confounded. Therefore, further studies should investigate the association between metamemory and source memory performance that would match comparison subjects in terms of age and education factors. It is also possible that the self-monitoring deficits observed in this study may be linked to particular symptoms of psychosis. For example, using a measure of the Positive and Negative Syndrome Scale (PANSS) (66), Gawęda et al. (9) observed correlations between imagined-actions-remembered-as-performed responses and hallucinations, and between these responses and general positive symptoms, but no correlation effect was observed in the severity of delusions [see also (67)]. Thus, in future studies the relationship between metacognitive processes induced by the PDW scale and the severity of symptoms in psychosis should be investigated. In fact, it is very interesting to take into account the prevalence of hallucinations and self-monitoring errors among healthy individuals and patients with psychiatric disorders other than schizophrenia (9, 68–70).

To sum up, as opposed to assessments of metacognition with the CR scale, patterns of metacognitive responses based on economic categorization resulted in better metacognition accuracy in both patients with schizophrenia and healthy controls. This has potential applications, most notably in that a single scale can be built to determine the metacognitive responses for healthy and schizophrenic groups without the need to develop specific scales for each group. Moreover, as expected, the diagnosis effect seems to affect performance in the task with practically no effect on measures of confidence.

As future research prospects, additional factors might need to be considered to fully understand the mechanism behind the accuracy of metacognition arising from the PDW scale. These factors might be linked to individual patient characteristics, such as medication type and dosage, or the time period since diagnosis. In particular, the reduction or discontinuation of treatment with antipsychotic drugs may significantly improve patients’ cognitive functions (71). From the application perspective, the construction of “economic” feedback to treat metacognition in schizophrenia in order to distinguish between true and false memories might be considered. Thus, this study demonstrates promising implications of using imaginary monetary categorization in modern health-care programs for treating metacognition in psychiatric populations. The feasibility and efficacy of metacognitive therapy in patients with schizophrenia have been demonstrated in several empirical reports (2, 72–76). However, one also should be aware of a recent meta-analysis indicating that there is no convincing empirical evidence for the unambiguous efficacy of MCT (77), since group or individualized MCT interventions show small or small-to-medium effect sizes on average. These relatively poor outcomes of MCT in the reduction of positive symptoms (e.g., delusions) are explained partially by abnormal data gathering and reasoning biases in patients with schizophrenia, who often are under stress and driven by negative mood (77). Nevertheless, it seems reasonable to apply our findings concerning the effects of monetary incentives on metacognition in therapeutic practice to make attempts to reduce cognitive bias in the population of patients with schizophrenia. Future empirical research will be needed to address the potential benefits of activating gambling strategies based on motivation and aversion to loss in treating metacognition in psychiatric populations.

## Conclusion

The main findings of our research can be summarized as follows. Firstly, patients diagnosed with schizophrenia showed poorer old/new recognition performance in the action memory task and, as opposed to healthy controls, they did not differ in false alarms and forgotten actions. Patients also committed more source memory errors because, as opposed to healthy controls, they were more likely to consider that imagined actions had been performed. Secondly, regardless of the group (patients with schizophrenia vs. healthy controls), evaluations of metacognition with the CR and PDW scales did not affect memory performance in the action memory task. Thirdly, compared to healthy controls, patients with schizophrenia showed a tendency to express higher confidence in self-monitoring errors (i.e., performed-as-imagined actions) when both measures of metacognition-measurement were employed, although the *p*-level of 0.054 implies that this effect might be debatable. Fourthly, there was no interaction effect between the type of metacognition measures (CR vs. PDW scale) and the diagnostic status of participants (patients with schizophrenia vs. healthy controls), thus indicating that both metacognition evaluation modes are the same for patients and healthy participants. This implies that patients and healthy participants are more likely to adjust their accuracy of metacognition when monetary incentives are at stake. In general, regardless of their diagnostic status, imaginary monetary loss induced by wagering diminishes participants’ confidence in their incorrect responses (i.e., memory errors and source memory errors) as compared to explicit metacognitive confidence in memory performance.

## Data Availability Statement

The datasets generated for this study are available on request to the corresponding author.

## Ethics Statement

The studies involving human participants were reviewed and approved by the Committee for Ethics in Empirical Studies with the Participation of People as Subjects of the SWPS University in Wroclaw. The patients/participants provided their written informed consent to participate in this study.

## Author Contributions

RS: research concept and design, data analysis and interpretation, writing the article, critical revision of the article, and final approval of article. EC: research concept and design, collection and assembly of data, data analysis and interpretation, writing the article, and final approval of article. AP: data analysis and interpretation, writing the article, and final approval of article. ŁG: writing the article, critical revision of the article, and final approval of article. JR: writing the article, critical revision of the article, and final approval of article.

## Funding

This research is supported by the National Science Center (Poland) and is funded under award number 2014/15/B/HS6/03834 to RS.

## Conflict of Interest

The authors declare that the research was conducted in the absence of any commercial or financial relationships that could be construed as a potential conflict of interest.

## Footnotes

1 Feeling of knowing and the tip-of-the-tongue experience (53) induce the expectation in participants that the required information is available in memory, although it is currently inaccessible despite their efforts. Hence, these metacognitive feelings potentially motivate participants to search their memory further and use control strategies (54, 55).
